# Upper urinary tract stone compositions: the role of age and gender

**DOI:** 10.1590/S1677-5538.IBJU.2019.0278

**Published:** 2020-01-13

**Authors:** Shu Wang, Yitian Zhang, Xin Zhang, Yuzhe Tang, Jianxing Li

**Affiliations:** 1 Department of Urology, Beijing Tsinghua Changgung Hospital, Beijing, China; 2 Division of Urology, Department of Surgery, University of Maryland School of Medicine, Baltimore, USA; 3 University of North Carolina at Chapel Hill, Chapel Hill, USA

**Keywords:** Urinary Calculi, Age Groups, Gender Identity

## Abstract

**Objective:**

To analyze the compositions of upper urinary tract stones and investigate their distributions in different gender and age groups.

**Materials and Methods:**

Patients diagnosed with upper urinary tract stone disease between December 2014 and March 2018 were retrospectively reviewed. Patient's age, gender, BMI, comorbidities, stone event characteristics, and compositions were collected, and proportions of stone components in different gender and age groups were analyzed.

**Results:**

A total of 1532 stone analyses were performed (992 from males and 540 from females). The mean age was younger in males (p <0.001). Males included more cases with larger BMI, hyperuricemia, and obesity, while females had more urinary tract infections. Multiple components were present in 61.8% of stones. Calcium oxalate (CaOx) (67.0%) was the most common component, followed by uric acid (UA) (11.8%), infection stone (11.4%), calcium phosphate (CaP) (8.0%), cystine (1.1%), brushite (0.4%), and 2, 8-dihydroxyadenine (0.2%). Men contributed with more CaOx stones than women at age 30-49 years (all p <0.01) and more UA stones at 30-59 years (all p <0.05). Women contributed with more infection stones than men in age groups 30-49 and 60-69 years (all p <0.05), and more CaP stones at 30-49 years. The prevalence peak was 50-59 years in men and 60-69 years in women. Both genders had the lowest prevalence in adolescence. Prevalence of UA stones increased while that of infection stones decreased with aging in both genders.

**Conclusions:**

Age and sex had a strong association with distribution of stone compositions in this Chinese cohort.

## INTRODUCTION

The prevalence of urolithiasis is increasing in both developed and developing countries. Studies have demonstrated that ethnicity, geographic region, and living conditions could have an infl uence on stone formation (1). A recent study based on the United States population demonstrated that demographic factors, especially gender and age, had potential effects on stone composition, reporting that younger women had more hydroapatite stones, whereas older individuals were more susceptible to uric acid stones (2). Other studies from Europe have confi rmed a higher prevalence of uric acid stone formation in the older population (3, 4).

However, most of these studies were from Western countries and focused on Caucasians. Relatively little has been reported about the function of age and gender on stone composition in the Chinese population, and these published studies were conducted in local hospitals in southern and eastern areas. As one of the largest stone management institutes in northern China, we reviewed a large cohort of stone analysis reports from the last 3 years and investigated the possible association of age and gender with stone composition from an epidemiological perspective.

## MATERIALS AND METHODS

### Data collection

Patients diagnosed with upper urinary stone disease in Beijing Tsinghua Changgung Hospital from December 2014 to March 2018 were included. Only data from patients whose stones were retrieved intraoperatively (ureteroscopy and percutaneous nephrolithotomy) and sent for analysis were collected. Patient's demographic information such as age, gender, body mass index (BMI), and comorbidities were gathered. We also included history of lithotripsy and urinary tract infection (UTI), stone location, surgical approaches for stone acquisition, and stone composition for evaluation of stone episodes.

### Stone composition analysis

All stone samples gathered from the operation were analyzed in the stone analysis laboratory by infrared spectroscopy. After the specimens were washed, dried, and pulverized, 1mg was mixed with 100-200mg of potassium bromide into a uniform powder, and then laminated into a test slice to be placed in an infrared spectrum automatic analysis system for detection. The principal component of the stone was recorded, the classification criteria for which were as follows (2, 5, 6): calcium oxalate (CaOx) stone refers to that with any kind of CaOx >50%; calcium phosphate (CaP) stone contains >50% CaP; infection stone contains >10% magnesium ammonium phosphate or ammonium urate; uric acid (UA) stone contains >50% UA; brushite, cystine, and other rare compositions. For example, if the stone was composed of 40% calcium oxalate monohydrate (COM), 30% calcium oxalate dehydrate (COD), and 30% CaP, it would be considered as a CaOx stone. If the sample was found to consist of 20% magnesium ammonium phosphate, 60% CaP, and 20% COM, it would be classified as an infection stone.

## STATISTICAL METHODS

Patients were divided into nine age groups by 10-year intervals. The prevalence of stone components was evaluated by two parameters: proportion and the prevalence ratio adjusted by the population of China census in 2017. The prevalence of stone in females at age 0-9 years was chosen as the reference. Statistical analysis was conducted using SPSS 22.0 for Windows (SPSS, Chicago, IL, USA). The proportions of categorical variables were analyzed using the Chi-square test and Fisher's exact test. Uni-and multivariate logistic regression analyses were performed to evaluate the risk factors contributing to stone composition conversion. P <0.05 was considered statistically significant.

## RESULTS

**Figure 1:**
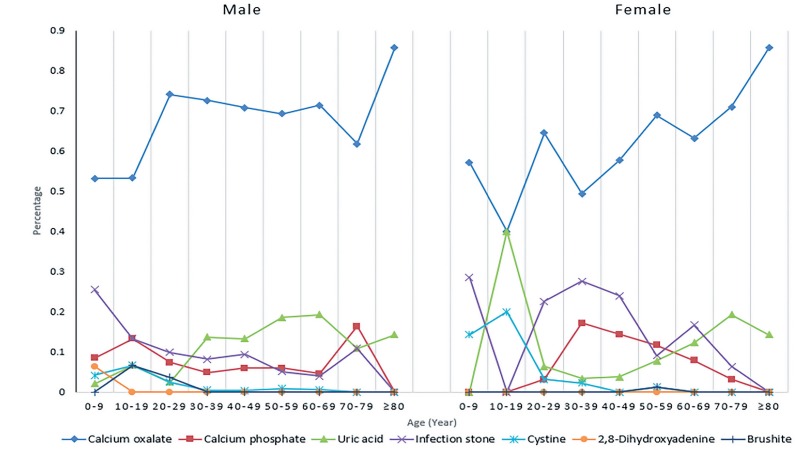
Proportions of stone compositions in different age groups and genders.

Between December 2014 and March 2018, 1532 stone analyses were performed, 992 of which were from males and 540 from females. The mean age was younger in males than in females (45.9 vs. 50.0 years, p <0.001). Males included more cases with larger BMI, hyperuricemia, and obesity (BMI ≥30kg/m2), while females had more UTIs (Table-1). More kidney stones and percutaneous nephrolithotomies (PCNL) were seen in females (p <0.001). A total of 948 (61.8%) stones were composed of multiple elements, to which women contributed much more than males (70.9% vs. 57.0%, p <0.001). CaOx (67.0%) was the most common component, followed by UA (11.8%), infection stone (11.4%), CaP (8.0%), cystine (1.1%), brushite (0.4%), and 2, 8-dihydroxyadenine (0.2%). The four most common stone compositions in men were CaOx stone (69.8%), UA stone (13.8%), infection stone (8.3%), and CaP stone (6.5%), while in women they were CaOx stone (62.0%), infection stone (17.1%), CaP stone (10.9%), and UA stone (8.1%) (Figure-1). The proportions of CaOx (p=0.002) and UA (p=0.002) were much higher in men than in women, whereas the proportions of infection stone (p <0.001) and CaP (p=0.002) were much higher in women.

**Table 1:** Characteristics of patients and stone compositions stratified by gender.

Parameters		Total number N=1532	Stones from Males N=992	Stones from Females N=540	p-value
	Age (year, mean±SD)	47.4±16.4	45.9±17.1	50.0±14.7	<0.001
	BMI (kg/m2)	25.9±3.4	27.4±3.2	24.9±3.5	0.011
**Comorbidities**					
	Hypertension	329 (21.5%)	219 (22.1%)	110 (20.4%)	0.473
	Diabetes	206 (13.4%)	128 (12.9%)	78 (14.4%)	0.398
	Hyperuricemia	213 (13.9%)	151 (15.2%)	62 (11.5%)	0.043
	Obesity (BMI .30kg/m2)	277 (18.1%)	200 (20.1%)	77 (14.3%)	0.004
**History of lithotripsy**					
	No	952 (62.1%)	607 (61.2%)	345 (63.9)	0.298
	ESWL	241 (15.7%)	152 (15.3%)	89 (16.5%)	0.552
	Ureteroscopy	153 (10.0%)	95 (9.6%)	58 (10.7%)	0.468
	PCNL	214 (14.0%)	138 (13.9%)	76 (14.1%)	0.930
	Open or laparoscopic nephrolithotomy	55 (3.6%)	41 (4.1%)	14 (2.6%)	0.122
	Urinary tract infection	547 (35.7%)	336 (33.9%)	211 (39.1%)	0.042
**Episode**					
	First	866 (56.5%)	555 (55.9%)	311 (57.6%)	0.535
	Recurrent	666 (43.5%)	437 (44.1%)	229 (42.4%)	
**Stone location**					
	Kidney	1128 (73.6%)	768 (77.4%)	460 (85.2%)	<0.001
	Ureter	304 (26.4%)	224 (22.6%)	80 (14.8%)	
**Surgery of stone acquisition**					
	Ureteroscopy	722 (47.1%)	493 (49.7%)	229 (42.4%)	0.006
	PCNL	810 (52.9%)	499 (50.3%)	311 (57.6%)	
**Composition**					
	Single	584 (38.2%)	427 (43.0%)	157 (29.1%)	<0.001
	Mixed	948 (61.8%)	565 (57.0%)	383 (70.9%)	
**Stone compositions**					
	Calcium oxalate	1027 (67.0%)	692 (69.8%)	335 (62.0%)	0.001
	Infection stone	175 (11.4%)	82 (8.3%)	93 (17.2%)	<0.001
	Calcium phosphate	123 (8.0%)	64 (6.5%)	59 (10.9%)	0.002
	Uric acid	181 (11.8%)	137 (13.8%)	44 (8.1%)	0.001
	Cystine	17 (1.1%)	10 (1.0%)	7 (1.3%)	/
	2.8-Dihydroxyadenine	3 (0.2%)	3 (0.3%)	0	/
	Brushite	6 (0.4%)	4 (0.4%)	2 (0.4%)	/

The age-specific prevalence ratios showed that the lowest prevalence for both males and females was in their adolescence, and the peak of prevalence was observed during the age of 50-59 years in men and 60-69 years in women (Table-2). Men presented more CaOx stones than women in the 30-49 years (all p <0.05), and more UA stones at age 30-59 years (p <0.05 for all). Women accounted for more infection stones than men between the ages of 30-49 and 60-69 years (all p <0.05), and more CaP stones at age 30-49 years (Table-3). The prevalence of UA stones was higher in both genders after their 40s.

**Table 2:** Age-specific prevalence ratios adjusted by the population of China census in 2017.

Age Groups (years)	Number of Patients		Population Percentage (Vintage 2017)		M/F Population Ratio	Age-specific Incidence Ratio	
	M	F	M	F		M	F
0-9	47 (3.1%)	7 (0.5%)	6.3%	5.5%	1.15	5.86	1 (Ref.)*
10-19	15 (1.0%)	5 (0.3%)	5.8%	5%	1.16	2.03	0.79
20-29	81 (5.3%)	31 (2.0%)	8.3%	7.5%	1.11	7.67	3.25
30-39	182 (11.9%)	87 (5.7%)	7.6%	7.1%	1.07	18.82	9.63
40-49	233 (15.2%)	104 (6.8%)	8.6%	8.2%	1.05	21.29	9.97
50-59	215 (14.0%)	154 (10.1%)	7.1%	6.9%	1.03	23.79	17.54
60-69	150 (9.8%)	114 (7.4%)	5%	5%	1.00	23.57	17.91
70-79	55 (3.6%)	31 (2.0%)	2.1%	2.2%	0.95	20.58	11.07
≥80	14 (0.9%)	7 (0.5%)	0.5%	1%	0.80	13.75	5.5
**Total**	**992 (64.8%)**	**540 (35.2%)**	**51.6%**	**48.4%**	**1.07**	**15.11**	**8.77**

* Reference: The prevalence of stone in females in age 0-9 years.

**Table 3:** Proportions of different stone compositions stratified by age groups.

	Total	0-9	10-19	20-29	30-39	40-49	50-59	60-69	70-79	≥80
Calcium oxalate	1027	29 (2.8%)	10 (1.0%)	80 (7.8%)	175 (17.0%)	225 (21.9%)	255 (24.8%)	179 (17.4%)	56 (5.5%)	18 (1.8%)
Male	692	25 (3.6%)	8 (1.2%)	60 (8.7%)	132 (19.1%)	165 (23.8%)	149 (21.5%)	107 (15.5%)	34 (8.1%)	12 (1.7%)
Female	335	4 (1.2%)	2 (0.6%)	20 (6.0%)	43 (12.8%)	60 (17.9%)	106 (31.6%)	72 (21.5%)	22 (6.6%)	6 (1.8%)
p-value		0.585	0.5	0.316	<0.001	0.018	0.923	0.159	0.393	0.753
Calcium phosphate	123	4 (3.3%)	2 (1.6%)	7 (5.7%)	24 (19.5%)	29 (23.6%)	31 (25.2%)	16 (13.0%)	10 (8.1%)	0
Male	64	4 (6.3%)	2 (3.1%)	6 (9.4%)	9 (14.1%)	14 (21.9%)	13 (20.3%)	7 (10.9%)	9 (14.1%)	0
Female	59	0	0	1 (1.7%)	15 (25.4%)	15 (25.4%)	18 (30.5%)	9 (15.3%)	1 (1.7%)	0
p-value		0.564	0.553	0.374	0.001	0.011	0.054	0.276	0.064	/
Uric acid	181	1 (0.6%)	3 (1.7%)	4 (2.2%)	28 (15.5%)	35 (19.3%)	52 (28.7%)	43 (23.8%)	12 (6.6%)	3 (1.7%)
Male	137	1 (0.7%)	1 (0.7%)	2 (1.5%)	25 (18.2%)	31 (22.6%)	40 (29.2%)	29 (21.2%)	6 (4.4%)	2 (1.5%)
Female	44	0	2 (4.5%)	2 (4.5%)	3 (6.8%)	4 (9.1%)	12 (27.3%)	14 (31.8%)	6 (13.6%)	1 (2.3%)
p-value		0.87	0.14	0.306	0.01	0.009	0.003	0.124	0.221	0.726
Infection stone	175	14 (8.0%)	2 (1.1%)	15 (8.6%)	39 (22.3%)	47 (26.9%)	25 (14.3%)	25 (14.3%)	8 (4.6%)	0
Male	82	12 (14.6%)	2 (2.4%)	8 (9.8%)	15 (18.3%)	22 (26.8%)	11 (13.4%)	6 (7.3%)	6 (7.3%)	0
Female	93	2 (2.2%)	0	7 (7.5%)	24 (25.8%)	25 (25.9%)	14 (15.1%)	19 (20.4%)	2 (2.2%)	0
p-value		0.591	0.553	0.076	<0.001	<0.001	0.134	<0.001	0.396	/
Others*	26	6 (23.1%)	3 (11.5%)	6 (23.1%)	3 (11.5%)	1 (3.8%)	6 (23.1%)	1 (3.8%)	0	0
Male	17	5 (29.4%)	2 (11.8%)	5 (29.4%)	1 (5.9%)	1 (5.9%)	2 (11.8%)	1 (5.9%)	0	0
Female	9	1 (11.1%)	1 (11.1%)	1 (11.1%)	2 (22.2%)	0	4 (44.4%)	0	0	0

* Others included cystine stone, 2, 8-dihydroxyadenine, and brushite.

We further analyzed stone conversions in different episodes. A total of 118 patients with two stone episodes and surgical removal of stones were identified in our center, of whom 48 (40.7%) had stone conversions in the second episode. Multivariate analysis showed that only CaP in the first episode (OR 14.178, 95%CI 2.655-75.702, p=0.002) was associated with stone conversion in the second episode, after controlling for age, gender, and the procedure for the first episode (Supplementary Tables).

## DISCUSSION

Urinary stone disease is a common disorder worldwide, causing severe socioeconomic and healthcare burden. Approximately 1 in 11 people in the United States will be affected by urinary stones during their lifetime (7). The incidence of urolithiasis in China has been increasing steadily during the last 20 years, mainly because of changing of lifestyles factors, especially diet (8). The most recent nationwide study revealed that about 1 in 17 adults in China has renal stones (9).

Urolithiasis is reported to be a male-dominant disease with an estimated male-to-female ratio ranging from 1.7:1 to 3:1 (10–12). We also observed a male dominance pattern with a ratio of 1.8 in our study. Stone compositions differed significantly between genders. Men presented more CaOx and UA stones than women, consistent with recent studies in other parts of China (13, 14). Males had a tendency to consume more animal meat and proteins, thus developing oversaturated CaOx and UA in the urine (15). It is worth noting that males had more cases with larger BMI, hyperuricemia, and obesity in our study population, which might be one reason for such a pattern.

The prevalence of infection stones among women in our study was two times of that in men (17.22% vs. 8.27%). Women are known to be at higher risk of developing UTIs, which in turn will elevate urinary pH and facilitate the growth of organisms containing urease (16). Younger females are observed to possess a higher urine pH (14), which could explain why in our study the prevalence of infectious stones was higher in women aged 30-49 years and lower in older age groups. Overall, the rate of infectious stones observed in our center was much higher than in any other centers in China. As a tertiary hospital, our institution manages more complex stone cases with severe infection and staghorn stones, which may lead to a selection bias. CaP, the second most common stone component in females, is associated with renal acidification defects, calcium or phosphate metabolic disturbance, and UTI (17). The theory that CaP is formed in alkaline urine (18) could support our finding of the disparity between genders because women generally possess urine of higher pH.

We observed component discrepancies among different age groups. Men submitted more CaOx stones than women at age 30-49 years, more UA stones at age 30-59 years, fewer infection stones at age 30-59 years, and fewer CaP stones at age 30-49 years. Although CaOx was the most common component in both genders, peaking at 30-69 years, differences have been observed between continents, with CaOx peaking at 40-50 years of age in Europe and 16-39 years in northern Africa. Wu et al. reported a peak age for CaOx stones at 19-40 years in southern China (13), whereas Yang et al. reported a prevalence peak of 30-50 years in eastern China (14). Ethnicity, different dietary preferences, and population composition may contribute collectively to create such disparity.

UA has been reported to have increased dramatically in recent years. Wu et al. reported that compared with 2003-2007, the number of UA stones in 2008-2012 doubled (3.83% vs. 6.94%) (13). In our study, UA contributed to 11.8% of all stones, which was almost the same as that reported by a recent study in China in 2019, with a UA stone prevalence of 11.1% (9). We also found that the prevalence of UA stones slowly increased among both genders with aging (2). UA stones have a close association with metabolic syndrome and renal insufficiency, which are more prevalent in older people than in the younger generation (19). Increased acidic urine observed in elderly patients can lead to renal proximal tubular injury and impaired tubular alkalization (20), predisposing older people to UA stones.

Studies have demonstrated that the metabolic changes after the menopause might have an influence on urine components, increasing the risk of stone formation in this population. Prochaska et al. reported a higher risk of renal stones in post-menopausal women (OR 1.27, 95% CI 1.08-1.46) (21). Zhao et al. demonstrated that postmenopausal women were predisposed to urinary stones with lower blood estrogen (22), and Heller et al. reported that postmenopausal women who were treated with estrogen had lower 24h calcium excretion and CaOx saturation (23). In our study, we did observe an increasing prevalence of stone disease in women after their 50s. As women reached their 70s and 80s the proportions of stone components become similar to those of men, in another way supporting the notion that the menopause might have an influence on stone formation. Based on our data, we were unable to conclude whether the menopause is an independent risk factor for stone formation, since details such as menstrual status, level of serum estrogen, urine metabolic analysis, and lifestyle and dietary changes in older females were not available in our study population.

Generally, the prevalence of urinary stones in children is much lower than that in adults, with reported rates of 2% to 5% (24). However, stone formation can precede a high rate of recurrence and considerable morbidity in children. In our study, incidences for both genders were relatively high in their first decade and decreased as they entered adolescence. Genetic and metabolic disorders are common in children with urinary stone diseases, with hypercalciuria and hypocitraturia being the most prevalent (24). In a study conducted in southern China, stones from 382 children included 78.8% CaOx, 10.7% infection stones, 9.4% cystine, and 1.1% UA, of which 97.5% of the patients manifested as low citrate (25). Differences have been observed among different areas within China. One study from eastern China reported a relatively high rate of CaOx and cystine stones, and a low rate of struvite stones in children (26), while a study in the northwest border region reported ammonium urate as the dominant stone component in the whole population (27). An early study in Argentina reported that 84.4% of the children with urinary stone disease had metabolic abnormalities, with hypercalciuria and hypocitraturia being the two major risk factors (24). Twenty-four-hour urine analysis and genetic tests are recommended strongly for children with early onset of urinary stone disease to evaluate possible metabolic and genetic disorders and target the treatment to reduce recurrence (28).

Drug-and food-induced urinary tract stones are much more common in children. In 2008, China experienced an event of melamine contamination of powdered-milk formula for infants (29). When the concentration of melamine from the formula was sufficiently high in the urine, it easily formed crystals or stones in the infants because of its low solubility (30). The stones analyzed during this crisis were proved to be composed of uric acid dihydrate and ammonium urate (26). It is incumbent on urologists to apply the new knowledge of such rare etiology to the improvement of medical care treatment of children.

The present study was one of the very few that investigated the effects of age and gender on stone compositions among the Chinese population. However, some limitations are apparent. First, although we compared the prevalence ratios between age groups adjusted by the population proportions, the actual prevalence of the urinary stone disease in the real world could not be calculated because the data were gathered from patients with urolithiasis. Second, all stone samples were retrieved from operations, leading to a bias against the existence of an unknown number of patients with asymptomatic stones or experiencing spontaneous stone passage away from the hospital. Third, our department admitted mainly adult patients, which could also introduce bias regarding the evaluation of children. Lastly, because of the retrospective study design, we were unable to gather information such as 24-hour urine analysis. It may be concluded that age and gender represent the epidemiological features, not the direct functional factor. Further well-designed multicenter studies are needed to better elucidate the mechanisms behind these differences.

## CONCLUSIONS

In conclusion, our study presented an analysis of stone compositions in a large population in northern China. Significant disparities of stone compositions existed among different genders and ages. Overall, males submitted more CaOx and UA stones whereas females were susceptible to CaP and infectious stones. Incidence of UA stones increased with aging while that of infection stones decreased. The underlying mechanism, including metabolic differences and the function of estrogen, needs to be investigated in the future.

## ETHICAL APPROVAL

The protocol was approved by the institutional ethics committee.

## SUPPLEMENTARY TABLES

[Table t4] [Table t5]

**Supplementary Table 1 t4:** Characteristics of patients with or without stone conversions in two episodes.

Variables		Total number N=118	Conversion N=43	No conversion N=75	P-value
**Gender**					
	Male	68 (57.6%)	22 (51.2%)	46 (61.3%)	0.282
	Female	50 (42.4%)	21 (48.8%)	29 (38.7%)	
Age (year, mean±SD)		47.3±14.7	46.7±15.5	47.7±14.2	0.617
**Prior procedure (first of the two episodes)**					
	Ureteroscopy	56 (47.5%)	20 (46.5%)	36 (48.0%)	0.876
	PCNL	62 (52.5%)	23 (53.5%)	39 (52.0%)	
Compositions (first of the two episodes)					
	Calcium oxalate	61 (51.7%)	17 (39.5%)	44 (58.7%)	0.004
	Infectious stone	24 (20.3%)	10 (23.2%)	14 (18.7%)	0.551
	Calcium phosphate	13 (11.0%)	11 (25.6%)	2 (2.7%)	<0.001
	Uric acid	14 (11.9%)	3 (7.0%)	11 (14.7%)	0.214
	Cystine	3 (2.5%)	1 (2.3%)	2 (2.7%)	/
	2.8-Dihydroxyadenine	1 (0.8%)	0	1 (1.3%)	/
	Brushite	2 (1.7%)	1 (2.3%)	1 (1.3%)	/

**Supplementary Table 2 t5:** Multivariate analysis of the predictors for stone composition conversion.

		Variables		Multivariate	
Variables		Odds Ratio (95% Confidence Interval)	p-value	Odds Ratio (95% Confidence Interval)	p-value
**Gender**					
	Male	1		1	
	Female	1.514 (0.710-3.229)	0.283	1.205 (0.526-2.760)	0.659
**Age (year)**					
	<40	1		1	
	40-70	0.690 (0.294-1.614)	0.392	0.651 (0.256-1.654)	0.367
	>70	0.436 (0.041-4.689)	0.493	0.642 (0.055-7.498)	0.724
**Prior procedure (first of the two episodes)**					
	Ureteroscopy	1		1	
	PCNL	0.803 (0.379-1.701)	0.566	1.081 (0.387-3.014)	0.882
**Compositions (first of the two episodes)**					
	Calcium oxalate	1		1	
	Infectious stone	1.849 (0.690-4.955)	0.222	1.629 (0.518-5.121)	0.404
	Calcium phosphate	14.235 (2.853-71.020)	0.001	14.178 (2.655-75.702)	0.002
	Uric acid	0.706 (0.175-2.845)	0.624	0.708 (0.173-2.901)	0.631
	Cystine	1.294 (0.110-15.221)	0.838	1.282 (0.102-16.096)	0.847
	2.8-Dihydroxyadenine	0.000	1.000	0.000	1.000
	Brushite	2.588 (0.153-43.760)	0.510	2.327 (0.129-41.841)	0.567
